# *In vivo* Emergence of Colistin Resistance in Carbapenem-Resistant *Klebsiella pneumoniae* Mediated by Premature Termination of the *mgrB* Gene Regulator

**DOI:** 10.3389/fmicb.2021.656610

**Published:** 2021-06-21

**Authors:** Yingying Kong, Chao Li, Hangfei Chen, Wei Zheng, Qingyang Sun, Xinyou Xie, Jun Zhang, Zhi Ruan

**Affiliations:** Department of Clinical Laboratory, Sir Run Run Shaw Hospital, Zhejiang University School of Medicine, Hangzhou, China

**Keywords:** *Klebsiella pneumoniae*, colistin resistance, *mgrB*, complementation, whole genome sequencing

## Abstract

Multidrug-resistant (MDR) *Klebsiella pneumoniae* is a severe threat to public health worldwide. Worryingly, colistin resistance, one of the last-line antibiotics for the treatment of MDR *K. pneumoniae* infection, has been increasingly reported. This study aims to investigate the emergence of evolved colistin resistance in a carbapenem-resistant *K. pneumoniae* isolate during colistin treatment. In this study, a pair of sequential carbapenem-resistant *K. pneumoniae* isolates were recovered from the same patient before and after colistin treatment, named KP1-1 and KP1-2, respectively. Antibiotic susceptibility testing was performed by the microdilution broth method. Whole genome sequencing was performed, and putative gene variations were analyzed in comparison of the genome sequence of both isolates. The bacterial whole genome sequence typing and source tracking analysis were performed by BacWGSTdb 2.0 server. Validation of the role of these variations in colistin resistance was examined by complementation experiments. The association between colistin resistance and the expression level of PhoP/PhoQ signaling system and its regulated genes was evaluated by quantitative real-time PCR (qRT-PCR) assay. Our study indicated that KP1-1 displayed extensively antibiotic resistant trait, but only susceptible to colistin. KP1-2 showed additional resistance to colistin. Both isolates belonged to Sequence Type 11 (ST11). The whole genome sequence analysis uncovered multiple resistance genes and virulence genes in both isolates. No plasmid-mediated *mcr* genes were found, but genetic variations in five chromosomal genes, especially the Gln30^∗^ alteration in MgrB, were detected in colistin-resistant isolate KP1-2. Moreover, only complementation with wild-type *mgrB* gene restored colistin susceptibility, with colistin MIC decreased from 32 to 1 mg/L. Expression assays revealed an overexpression of the *phoP*, *phoQ*, and *pmrD* genes in the *mgrB*-mutated isolate KP1-2 compared to the wild-type isolate KP1-1, confirming the MgrB alterations was responsible for increased expression levels of those genes. This study provides direct *in vivo* evidence that Gln30^∗^ alteration of MgrB is a critical region responsible for colistin resistance in *K. pneumoniae* clinical strains.

## Introduction

Carbapenem resistance in *Klebsiella pneumoniae* has increased worldwide, mainly due to the rapid dissemination of antimicrobial-resistant bacteria and carbapenem overconsumption, thus limiting the effectiveness of therapeutic regimens (Tzouvelekis et al., 2012; van Duin and Doi, 2017). Polymyxins (polymyxin B and colistin) are considered as the last-resort antibiotics to treat infections caused by carbapenem-resistant *K. pneumoniae* (Giamarellou, 2016; Poirel et al., 2017). Colistin initially exhibited robust antibacterial activity for carbapenem-resistant *K. pneumoniae*, however, the emergence of colistin-resistant isolates has been reported repeatedly as its use expanded (Cannatelli et al., 2013; Jayol et al., 2014; Olaitan et al., 2014a; Aires et al., 2016; Giamarellou, 2016; Haeili et al., 2017; Hamel et al., 2020).

In *K. pneumoniae*, resistance to polymyxins is mostly mediated by adding 4-amino-4-deoxy-L-arabinose (L-Ara4N) and/or phosphoethanolamine (PEtN) to the lipid A moiety of lipopolysaccharide (LPS), which reduces the affinity between polymyxins and LPS (Olaitan et al., 2014b; Poirel et al., 2017). This modification can be regulated by the PhoQ/PhoP and PmrAB signaling systems, which regulate the expression of *pmrCAB* and *pmrHFIJKLM* operons responsible for modification of lipid A (Jayol et al., 2014; Olaitan et al., 2014b; Poirel et al., 2017). MgrB is a small regulatory transmembrane protein and exerts negative feedback on the PhoQ/PhoP signaling system (Lippa and Goulian, 2009). Thus, genetic alterations of MgrB have been proved to be responsible for colistin resistance (Cannatelli et al., 2013, 2014; Poirel et al., 2015; Aires et al., 2016; Hamel et al., 2020). Moreover, plasmid mediated mobile colistin resistance (*mcr* gene) has been reported as a transmissible resistance mechanism in Enterobacteriaceae, including *K. pneumoniae* (Liu et al., 2016; Caniaux et al., 2017; Ga et al., 2019).

In this study, we investigated the genomic variations between a paired colistin-susceptible and -resistant *K. pneumoniae* isolates consecutively recovered from a single patient, and demonstrated the mechanism responsible for the emergence of high-level resistance to colistin during *in vivo* treatment.

## Materials and Methods

### The Patient and Isolates

A 66-year-old female patient was hospitalized, in a tertiary hospital in Hangzhou, Zhejiang province, China, in 2019, with symptoms of pyosepticemia, pancreatic malignancy, leukopenia, thrombocytopenic purpura, hepatic failure, and renal insufficiency. Initially, the patient received tigecycline by intravenous injection. Isolates were cultured from the inpatient during her hospitalization. The first isolate KP1-1, cultured form the blood sample of the inpatient within 24 h after admission, displayed extensively antibiotic resistance (including tigecycline) but susceptible only to colistin. The antibiotic therapeutic strategy was then adjusted to that of intravenous colistin (500,000 Unit every 8 h for the first 4 days and every 12 h for the following days). After received colistin treatment for 9 days, the second isolate KP1-2 was cultured from the stool sample with a colistin MIC of 32 mg/L. At the end of the treatment period, the inpatient expired from pyosepticemia and pancreatic malignancy. The isolates were identified by VITEK 2 (bioMérieux, Marcy-l’Étoile, France) and Matrix-assisted laser desorption/ionization-time-of-flight mass spectrometry (MALDI-TOF-MS, Bruker, Billerica, MA, United States).

### Antimicrobial Susceptibility Testing

Antimicrobial susceptibility testing was performed by the microdilution broth method for the following antimicrobial agents: aztreonam, fosfomycin, ertapenem, ceftazidime, cefepime, cefoperazone-sulbactam, cefoxitin, levofloxacin, ciprofloxacin, amikacin, tetracycline, minocycline, tigecycline, colistin, cefotaxime, meropenem, imipenem, gentamicin, trimethoprim-sulfamethoxazole, piperacillin, and piperacillin-tazobactam. The results were interpreted according to Clinical and Laboratory Standards Institute (CLSI) guidelines, except for tigecycline and colistin, which were interpreted according to the European Committee on Antimicrobial Susceptibility Testing (EUCAST) guidelines. *Escherichia coli* (ATCC 25922) were used as quality control strains for antimicrobial susceptibility testing.

### Whole-Genome Sequencing

Genomic DNA was extracted using a QIAamp DNA MiniKit (Qiagen, Valencia, CA, United States) following the manufacturer’s instructions. The bacterial genome was fragmented by sonication using a Covaris M220 sonicator (Covaris, Woburn, MA, United States) and the sheared DNA fragments were then used to prepare a shotgun paired-end library with an average insert size of 350 bp via a TruSeq DNA Sample Prep kit (Illumina, San Diego, CA, United States). The prepared library was sequenced using the Illumina NovaSeq 6000 platform (Illumina, San Diego, CA, United States) through the 150 bp paired-end protocol. The short reads were assembled using Unicycler v0.4.8 software (Wick et al., 2017).

The genome annotation was conducted using the NCBI Prokaryotic Genome Annotation Pipeline (PGAP) (Tatusova et al., 2016). Antibiotic resistance genes, virulence genes, and plasmid replicons were queried using ABRicate 1.0.1 in tandem with ResFinder 4.1, CARD 2020, VFDB 2019, and PlasmidFinder 2.1 databases, with a 90% threshold for gene identification and a 60% minimum length to respective database entries. With the genomic sequence of the first isolate KP1-1 as the reference, the reads of the second isolate KP1-2 was mapped against that of KP1-1 using CLC Genomics Workbench 12. The gene variations, including single nucleotide polymorphisms (SNPs) and insertion and deletion mutations were predicted, and the variations were verified by PCR and Sanger sequencing. *In silico* multilocus sequence typing (MLST) analysis and bacterial source tracking using core genome MLST (cgMLST) strategy were performed using BacWGSTdb 2.0 server (Ruan and Feng, 2016; Ruan et al., 2020; Feng et al., 2021). The phylogenetic relationship between *K. pneumoniae* KP1-1, KP1-2 and a total of 631 publicly available ST11 *K. pneumoniae* isolates recovered from China were analyzed.

### Complementation Assays

The high-copy plasmid pCR2.1-Hyg, constructed by inserting a hygromycin-resistant gene into the HindIII site of pCR2.1, was used as a genetic vector. *Escherichia coli* DH5α was used as the host for recombinant plasmids. The differential genes were amplified from the colistin-susceptible isolates KP1-1 using the primers shown in Table 1. The purified amplified fragments were, respectively, cloned into the plasmid pCR2.1-Hyg using the ClonExpress II One Step Cloning Kit (Vazyme, Nanjing, China). Then, the recombinant plasmids were separately transformed into the *E. coli* DH5α for amplification, and eventually introduced into the colistin-resistant isolates KP1-2 by electroporation. The plasmid pCR2.1-Hyg was also transformed into KP1-2 as blank control. Electro-transformants were selected on Mueller-Hinton agar supplemented with 40 g/mL of hygromycin.

**TABLE 1:** Primers used in this study.

**Primer name**	**Sequence (5′→ 3′)**	**Amplicon size (bp)**	**Reference or source**
**Conventional PCR**			
mgrB-SpeI-F	acactggcggccgttactagtAACACGTTTTGAAACAAGTCGATG	373	This study
mgrB-KpnI-R	tgactgggtcatggtggtaccCACCACCTCAAAGAGAAGGCG		This study
aroP-SpeI-F	acactggcggccgttactagtATGGAAGGTCAACAGCACGG	1413	This study
aroP-KpnI-R	tgactgggtcatggtggtaccTTATTGTGCTTTTATGGTGGCG		This study
fructokinase-SpeI-F	acactggcggccgttactagtATGAATGGAAAAATCTGGGTACTCG	924	This study
fructokinase-KpnI-R	tgactgggtcatggtggtaccTCATGGCGCCTTTGGCGG		This study
lysR-SpeI-F	acactggcggccgttactagtATGAAACTGCGTCATCTGGAAAT	957	This study
lysR-KpnI-R	tgactgggtcatggtggtaccTTACCCCAGCGGCGCAAT		This study
PTS system-SpeI-F	acactggcggccgttactagtATGAGTAAAGTGATCGATTCGCTTG	1401	This study
PTS system-KpnI-R	tgactgggtcatggtggtaccTCAGAATTTCAGTGCGTTAGCG		This study
**qRT-PCR**			
phoP_F	ATTGAAGAGGTTGCCGCCCGC	136	6
phoP_R	GCTTGATCGGCTGGTCATTCACC		
phoQ_F	ATATGCTGGCGAGATGGGAAAACGG	138	6
phoQ_R	CCAGCCAGGGAACATCACGCT		
pmrA_F	TACGCCGAAAGAGTATGCCC	170	This study
pmrA_R	GGATCCGCGATTTGCCAATC		This study
pmrB_F	TGC CAG CTG ATA AGC GTC TT	95	10
pmrB_R	TTC TGG TTG TTG TGC CCT TC		
pmrC_F	GCG TGA TGA ATA TCC TCA CCA	116	10
pmrC_R	CAC GCC AAA GTT CCA GAT GA		
pmrD_F	GAT CGC AGA GAT TGA AGC CT	120	10
pmrD_R	GCG TTG CGG ATC TTC AAA GT		
pmrE_F	GCA TAC CGT AAT GCC GAC TA	119	10
pmrE_R	GGG TTG ATC TCT GTG ACA TC		
pmrK_F	AGT ATC GGT CAG TGG CTG TT	123	10
pmrK_R	CCG CTT ATC ACG AAA GAT CC		
mgrB_F	CCTGTTGCTGTGGACTCAGA	73	This study
mgrB_R	AGTGCAAATGCCGCTGAAAA		This study
rpsL_F	CCGTGGCGGTCGTGTTAAAGA	109	6
rpsL_R	GCCGTACTTGGAGCGAGCCTG		

### Transcriptional Analysis by Quantitative Real-Time PCR (qRT-PCR)

Expression levels of *phoP*, *phoQ*, *pmrA*, *pmrB*, *pmrC*, *pmrD*, *pmrE*, *pmrK*, and *mgrB* genes were determined by qRT-PCR with the primers listed in Table 1. Total RNA was extracted from the mid-log phase bacterial culture using the PureLink^TM^ RNA Mini Kit (Thermo Fisher Scientific, Waltham, MA, United States) and treated with RNase-free DNase I (Takara Biotechnology, Dalian, China) to remove genomic DNA contamination. Then, the corresponding cDNAs were generated from 500 ng of RNA using the HiFiScript cDNA Synthesis Kit (CWBio, Beijing, China) according to the manufacturer’s instructions. qRT-PCR was performed using MagicSYBR Mixture (CWBio, Beijing, China) on the Applied Biosystems^TM^ QuantStudio^TM^ 1 (Thermo Fisher Scientific, Waltham, MA, United States). Thermal cycling conditions were as follows: 95°C for 30 s for enzyme activation, followed by 40 cycles of denaturation at 95°C for 5 s and annealing at 60°C for 30 s. The relative gene expression levels were determined using the comparative threshold cycle (ΔΔC_t_) method with *rpsL* gene normalization. The experiments were performed in triplicate and repeated three times.

### Accession Number

The draft genome sequence of *K. pneumoniae* KP1-1 and KP1-2 were deposited in the NCBI GenBank database under the accession numbers JAERIE000000000 and JAERIF000000000.

## Results

### Isolate Characterizations

The minimum inhibitory concentrations (MICs) of the two isolates to 21 antibiotics were shown in Table 2. Antimicrobial susceptibility testing showed that the first isolate KP1-1 was extensively drug-resistant, including aztreonam, ertapenem, ceftazidime, cefepime, cefoperazone-sulbactam, cefoxitin, levofloxacin, ciprofloxacin, amikacin, tetracycline, minocycline, tigecycline, cefotaxime, meropenem, imipenem, gentamicin, trimethoprim-sulfamethoxazole, piperacillin, and piperacillin-tazobactam. However, it remains susceptible to colistin (MIC < 0.03 mg/L). After the colistin therapy for 9 days, the second isolate KP1-2 was recovered with an increased colistin MIC of 32 mg/L.

**TABLE 2:** MICs of the *K. pneumoniae* KP1-1 and KP1-2 to different antimicrobial agents.

**Isolate**	**MIC (mg/L) ^*a*^**																				
	**ATM**	**FOF**	**ETP**	**CAZ**	**FEP**	**SCF**	**FOX**	**LVX**	**CIP**	**AMK**	**TET**	**TGC**	**MH**	**CST**	**CTX**	**MEM**	**IPM**	**GEN**	**SXT**	**PRL**	**PRL/TZP**
KP1-1	32	16	>256	>256	256	>256	256	64	128	>256	>256	8	64	<0.03	64	128	128	>256	16	>512	>512
KP1-2	32	32	>256	256	256	>256	128	128	128	>256	>256	8	64	32	128	128	128	>256	16	>512	512

*^*a*^ATM, aztreonam; FOF, fosfomycin; ETP, ertapenem; CAZ, ceftazidime; FEP, cefepime; SCF, cefoperazone-sulbactam; FOX, cefoxitin; LVX, levofloxacin; CIP, ciprofloxacin; AMK, amikacin; TET, tetracycline; TGC, tigecycline; MH, minocycline; CST, colistin; CTX, cefotaxime; MEM, meropenem; IPM, imipenem; GEN, gentamicin; SXT, trimethoprim-sulfamethoxazole; PRL, piperacillin; PRL/TZP, piperacillin-tazobactam.*

### Genomic Characteristics

**FIGURE 1:**
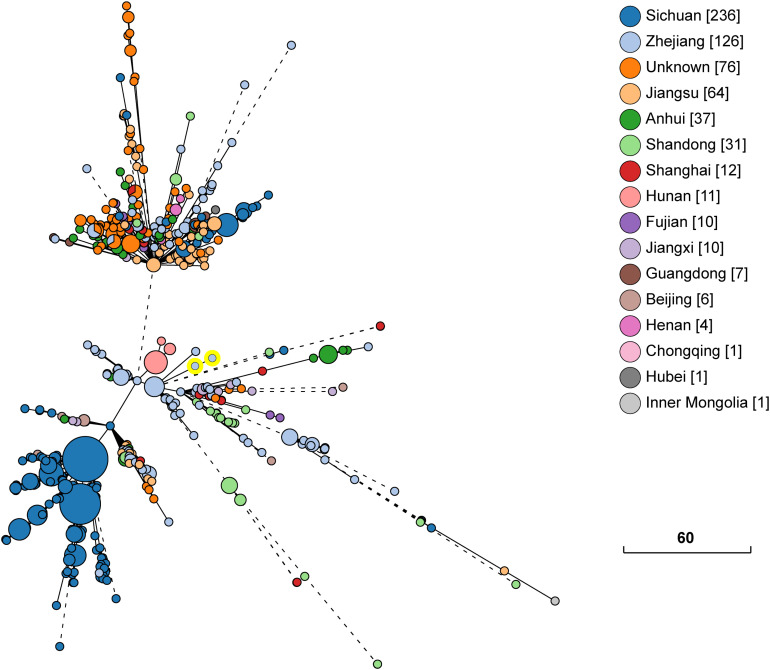
Phylogenetic relationship between *K. pneumoniae* KP1-1, KP1-2 and a total of 631 ST11 *K. pneumoniae* strains retrieved from NCBI GenBank. The lines connecting the circles indicate the clonal relationship between different isolates. The scale bar represents a pairwise allelic difference of core genome multilocus sequence typing (cgMLST) loci. The number of isolates from each province is given in square brackets. The solid line indicates the pairwise allelic differences of two isolates is less than 50 alleles. Branches longer than 50 allelic differences are proportionally shortened and are represented by dashed lines. The two yellow circles indicate *K. pneumoniae* KP1-1, KP1-2.

The whole genome sequence data analysis classified isolates KP1-1 and KP1-2 to the same Sequence Type 11 (ST 11). Both KP1-1 and KP1-2 harbored multiple antimicrobial resistance genes, including aminoglycosides (*aadA2* and *rmtB*), β-lactams (*bla*_CTX–M–65_, *bla*_KPC–2_ and *bla*_TEM–1B_), fluoroquinolones (*qnrS1*), fosfomycin (*fosA*), phenicols (*catA2*), sulfonamides (*sul2*), tetracyclines [*tet*(A)], and trimethoprim (*dfrA14*). Moreover, we also identified several virulence genes, including aerobactin (*iutA*, *iucA*, *iucB*, *iucC*, and *iucD*), hypermucoviscosity (*rmpA* and *rmpA2*), and yersiniabactin (*ybtA*, *ybtE*, *ybtP*, *ybtQ*, *ybtU*, *ybtT*, and *ybtX*). Phylogenetic analysis indicated that the majority of ST11 *K. pneumoniae* isolates in NCBI GenBank database were recovered from Sichuan and Hangzhou, and the most closely related strain to *K. pneumoniae* KP1-1 and KP1-2 is L20, another ST11 strain previously recovered from a human feces sample in Hangzhou in the year 2016, which differed by only 15 cgMLST loci (Figure 1).

### Genetic Variations in Colistin-Susceptible and -Resistant Isolates

Regarding to the colistin resistance, no plasmid-mediated genes (*mcr-1* to *mcr-10*) were found in isolate KP1-2. To explain the mechanism of colistin resistance, the raw sequence reads of KP1-1 and KP1-2 were mapped and putative variations were identified in seven genes (Table 3). Compared with the colistin-susceptible KP1-1, a premature stop codon was detected in the *mgrB* gene at the position 88 (C88T) in isolate KP1-2, leading to a non-sense mutation in the amino acid sequence for glutamine to stop at position 30 of the protein (Gln30^∗^). The full-length MgrB protein, which is 47 amino acids in wild type isolates, was therefore only 29 amino acids long in isolate KP1-2.

**TABLE 3:** Genetic alterations between the isolates KP1-1 and KP1-2.

**Genes**	**Annotation**	**Genotype**	**Genetic alterations**
*mgrB*	PhoP/PhoQ regulator MgrB	Non-sense mutation	C88T (Gln30*)
*aroP*	Aromatic amino acid transporter AroP	Missense variant	A689T (Glu230Val)
*chbC*	PTS N,N′-diacetylchitobiose transporter subunit IIC	Missense variant	C413T (Ala138Val)
*lysR*	LysR family transcriptional regulator	Missense variant	G770A (Cys257Tyr)
–	Aminoimidazole riboside kinase	Frame shift variant	714dupC (Ala239fs)
–	Fimbrial biogenesis outer membrane usher protein	Synonymous variant	T1641C (Ala547Ala)
*smvA*	Methyl viologen resistance protein	Synonymous variant	A711C (Thr237Thr)

Moreover, Missense variants were detected in aromatic amino acid transporter AroP (A689T, Glu230Val), PTS N,N’-diacetylchitobiose transporter subunit ChbC (C413T, Ala138Val), transcriptional activator protein LysR (G770A, Cys257Tyr), and a frameshift variant was detected in aminoimidazole riboside kinase (714dupC, Ala239fs). Synonymous variants were found in fimbrial biogenesis outer membrane usher protein (T1641C) and Methyl viologen resistance protein SmvA (A711>C).

### Complementation Experiments

The significance of the non-silent alterations of the five genes detected in colistin-resistant KP1-2 was determined by complementation experiments. The introduction of PCR2.1-Hyg-*mgrB*, which carries a cloned copy of the KP1-1 *mgrB* gene along with part of its flanking sequence, in isolate KP1-2 was able to restore susceptibility to colistin, with MIC decreased from 32 to 1 mg/L (Table 4). However, complementation with the rest four functional genes did not alter colistin susceptibility in KP1-2 isolates. This indicates that colistin resistance is not related to these alterations in the other four genes. On the contrary, transformed with the PCR2.1-Hyg, minor change of colistin MIC was found in KP1-2 (MIC decreased to 16 mg/L).

**TABLE 4:** The transformants of differential genes and the corresponding MICs of colistin.

**Strain**	**Colistin MIC (mg/L)**
KP1-1	<0.03125
KP1-2	32
KP1-2 (PCR2.1-Hyg)	16
KP1-2 (PCR2.1-Hyg-mgrB)	1
KP1-2 (PCR2.1-Hyg-aroP)	32
KP1-2 (PCR2.1-Hyg-fructokinase)	32
KP1-2 (PCR2.1-Hyg-lysR)	32
KP1-2 (PCR2.1-Hyg-pts system)	32

### Gln30^∗^ Substitution in MgrB Associated With *phoPQ* and *pmrD* Overexpression

Expression levels of the *phoPQ* operon and *pmr* genes were analyzed to assess the impact of the Gln30^∗^ substitution in MgrB. Analysis of *phoP*, *phoQ*, and *pmrD* transcription by qRT-PCR revealed a two- to three- fold increase in KP1-2 carrying an inactivated *mgrB* allele in comparison with KP1-1 and with the *mgrB* mutants complemented with a cloned copy of wild-type *mgrB* (Table 5). However, there was no upregulation in the expression of *pmrA, pmrB, pmrC, pmrE*, and *pmrK* genes. Interestingly, significant upregulation of *mgrB* was observed for KP1-2 (5-fold) and KP1-2 complemented with wild-type *mgrB* (10-fold).

**TABLE 5:** Colistin MICs and the expression levels of *phoP, phoQ, pmrA, pmrB, pmrC, pmrD, pmrE, pmrK*, and *mgrB* genes of *K. pneumoniae* KP1-1, KP1-2, and of the corresponding transformants carrying either the PCR2.1-Hyg or PCR2.1-Hyg-mgrB plasmids^*a*^.

**Stain**	**Chromosomal *mgrB* status**	**Colistin MIC (μg/mL)**			**Relative expression level (mean ± SD)**						
			***phoP***	***phoQ***	***pmrA***	***pmrB***	***pmrC***	***pmrD***	***pmrE***	***pmrK***	***mgrB***
KP1-1	WT	<0.03125	1	1	1	1	1	1	1	1	1
KP1-2	Premature termination	32	1.98 ± 0.18	3.25 ± 0.05	0.64 ± 0.03	0.57 ± 0.05	0.56 ± 003	2.26 ± 0.07	0.61 ± 0.05	0.96 ± 0.03	5.09 ± 0.22
KP1-2 (PCR2.1-Hyg)	Premature termination	16	1.93 ± 0.15	2.59 ± 0.17	0.76 ± 0.03	0.99 ± 0.08	1.14 ± 0.14	2.09 ± 0.11	0.98 ± 0.03	1.38 ± 0.10	4.9 ± 0.23
KP1-2 (PCR2.1-Hyg-mgrB)	Premature termination	1	1.07 ± 0.05	0.76 ± 0.02	0.70 ± 0.01	0.74 ± 0.02	1.12 ± 0.02	1.14 ± 0.10	0.98 ± 0.10	0.71 ± 0.07	10.43 ± 0.12

*^*a*^The expression levels for the *phoP*, *phoQ*, *pmrA*, *pmrB*, *pmrC*, *pmrD*, *pmrE*, *pmrK*, and *mgrB* genes were normalized against the value obtained with colistin-susceptible strain KP1-1.*

## Discussion

Over the past decade, increased rates of resistance to antibiotics in *K. pneumoniae* has been reported worldwide (Olaitan et al., 2014a; Giamarellou, 2016; van Duin and Doi, 2017). Colistin has regained a significant part of the therapeutic regimen for treatment of infection caused by carbapenem-resistant bacteria. However, it rapidly developed resistance due to the frequent use in clinical settings, becoming a major public health concern (Olaitan et al., 2014a; Giamarellou, 2016). In the present study, we monitored the evolved colistin resistance in a 66-year-old female inpatient infected with carbapenem-resistant *K. pneumoniae* during colistin treatment. Our data provide the direct evidence that genetic evolution in the *mgrB* gene can lead to a high level of colistin resistance and cause treatment failure.

Colistin resistance is mainly mediated by chromosome or horizontal gene transfer. Chromosomal mutations in two-component systems (PmrA/PmrB and PhoP/PhoQ) and genes regulating these systems can lead to colistin resistance in *K. pneumoniae* (Olaitan et al., 2014a,b; Poirel et al., 2017). Moreover, plasmid-mediated *mcr* genes, which encodes a phosphoethanolamine transfer enzyme, have been identified to confer resistance to colistin via horizontal gene transfer (Liu et al., 2016; Caniaux et al., 2017; Ga et al., 2019). None of the plasmid encoded *mcr-1* to *mcr-10* genes were detected in KP1-2, which demonstrating that the colistin resistance is mediated by chromosomally encoded mechanisms.

By mapping the whole genome sequences of KP1-1 and KP1-2, five genes with non-silent alterations were exhibited in colistin-resistant KP1-2, including inactivated *mgrB* gene mediated by premature termination. MgrB, a small transmembrane protein with 47 amino acids, mediates potent negative feedback on the PhoQ/PhoP regulatory system, which regulates genes implicated in the LPS modifications and colistin resistance (Lippa and Goulian, 2009; Olaitan et al., 2014b; Poirel et al., 2017). Until now, the insertion of IS elements (especially the IS*5*-like element), non-sense mutations, and missense mutations have recently been reported in colistin resistance in *K. pneumoniae* isolates in diverse clinical and non-clinical isolates (Cannatelli et al., 2013, 2014; Olaitan et al., 2014a; Poirel et al., 2015; Aires et al., 2016; Haeili et al., 2017; Hamel et al., 2020). Among the above genetic variations, insertional inactivation of *mgrB* by IS elements, especially IS5-like elements, seemingly to be the most common mechanism of *mgrB* variation. Regarding the missense mutations, genetic alterations in MgrB, including Q30stop and C28stop, have been identified to be responsible for colistin resistance in *K. pneumoniae* isolates (Olaitan et al., 2014a; Aires et al., 2016). We suppose that premature termination within *mgrB*, found in the present study, result in MgrB inactivation and therefore lead to PhoP/PhoQ activation which in turn activates the PmrA/PmrB response regulator.

Complementation experiments showed that only transformation of wild *mgrB* gene had the ability to restore colistin susceptibility in KP1-2. This result agrees with that *mgrB* disruptions and mutations represent a strong association with colistin resistance mechanism in *K. pneumoniae* (Cannatelli et al., 2013, 2014; Olaitan et al., 2014a; Poirel et al., 2015; Aires et al., 2016; Haeili et al., 2017; Hamel et al., 2020). The Gln30^∗^ substitution, has been reported in several studies in different countries, found in the KP1-2 reinforced the hypothesis that position C88 in the *mgrB* (codon 30 in protein) is a critical region, which is prone to mutate upon colistin treatment (Olaitan et al., 2014a; Poirel et al., 2015; Aires et al., 2016; Haeili et al., 2017). Compared with the colistin resistance resulting from the *mcr* genes and two-component systems, the inactivation of MgrB leads to a higher level of colistin resistance (Olaitan et al., 2014a; Liu et al., 2016; Ga et al., 2019). In the current study, MgrB variation conferring colistin resistance occurred in a successful pandemic clone ST11, which will likely cause global presence of pan-drug-resistant *K. pneumoniae* and need continuous monitor.

The disruption of *mgrB* results in the activation of PhoP/PhoQ signaling system, which is known to indirectly activate the PmrA/PmrB via PmrD (Lippa and Goulian, 2009; Olaitan et al., 2014b). The activation of the PmrA/PmrB leads to the upregulation of *pmrCAB* and *pmrHFIJKLM-pmrE* operons that transfer of PEtN and L-Ara4N cationic groups to the LPS, which is responsible for the acquisition of colistin resistance in *K. pneumoniae* (Olaitan et al., 2014b; Poirel et al., 2017). Thus, it is generally accepted that loss of MgrB activates the cross-regulation of PhoPQ-PmrD-PmrAB signal transduction pathway in *K. pneumoniae*. However, activation of PhoP/PhoQ through *mgrB* mutation dose not significantly activate the production of PmrA/PmrB and confers colistin resistance. Therefore, PhoP/PhoQ activation alone is able to confer colistin resistance even without any additional effects caused by PmrA/PmrB activation (Cheung et al., 2020). In our study, we observed a signification association between colistin resistance, attributed to Gln30^∗^ substitution in MgrB, and upregulation of *phoPQ* operon and *pmrD* gene despite there being no upregulation of *pmrHFIJKLM* and *pmrCAB* operons. This can be explained by the fact that some unexplained mechanisms other than *pmrHFIJKLM* and *pmrCAB* might be involved in mediating colistin resistance in *K. pneumoniae*, which warrants further investigation.

In conclusion, our findings identified that Gln30^∗^ substitution in MgrB is responsible for the upregulation of PhoP/PhoQ signaling system and of the *pmrD* gene that confers colistin resistance in *K. pneumoniae*. To the best of our knowledge, this is the first report to provide direct *in vivo* evidence that the alteration of MgrB confers colistin resistance in a carbapenem-resistant *K. pneumoniae* isolate in China.

## Data Availability Statement

The datasets presented in this study can be found in online repositories. The names of the repository/repositories and accession number(s) can be found in the article/supplementary material.

## Author Contributions

ZR, JZ, and XX designed the experiments. YK, CL, and HC performed the experiments. ZR, WZ, and QS analyzed the data. YK and ZR wrote the manuscript. All authors read and approved the final manuscript.

## Conflict of Interest

The authors declare that the research was conducted in the absence of any commercial or financial relationships that could be construed as a potential conflict of interest.
